# Clinical efficacy of general anesthesia versus local anesthesia for percutaneous transforaminal endoscopic discectomy

**DOI:** 10.3389/fsurg.2022.1076257

**Published:** 2023-01-06

**Authors:** Zhihua Wu, Jiahui He, Huantong Cheng, Shaohao Lin, Peng Zhang, De Liang, Xiaobing Jiang, Jianchao Cui

**Affiliations:** ^1^ Department of Spinal Surgery, First Affiliated Hospital of Guangzhou University of Chinese Medicine, Guangzhou, China; ^2^ The First Clinical Medical College, Guangzhou University of Chinese Medicine, Guangzhou, China

**Keywords:** lumbar disc herniation, percutaneous transforaminal endoscopic discectomy, local anesthesia, general anesthesia, pain management

## Abstract

**Objective:**

Local anesthesia (LA) is recommended for percutaneous transforaminal endoscopic discectomy(PTED), but satisfactory pain management is not mostly achieved. The goal of this study was to examine the clinical efficacy of PTED for lumbar disc herniation when performed under local anaesthetic vs. general anesthesia (GA).

**Methods:**

From August 2018 to August 2020, the clinical data of 108 patients treated with PTED were retrospectively evaluated and separated into two groups: LA and GA based on the anesthesia method. General information and clinical outcomes of patients were included. Visual analog scale (VAS) and Oswestry disability index (ODI) were recorded before operation, 1 week after operation, and 1 year after operation. In addition, VAS for back pain and leg pain on the second postoperative day were also recorded.

**Results:**

We divided the patients into two groups: 72 in LA and 36 in GA. There were no significant differences in gender, age, course of disease, body mass index, surgical segment, duration of operation, intraoperative bleeding, time of fluoroscopy, length of hospital stay, total hospitalization cost reoperation, surgical satisfaction, Macnab satisfaction, complications, preoperative and 1 year postoperatively VAS for back pain and leg pain and ODI, VAS for leg pain on the second day and 1 week postoperatively between the two groups (*P *> 0.05). VAS for back pain in GA group on the second day postoperatively, as well as the VAS for back pain and ODI at one week postoperatively, were better than those in LA group (*P *< 0.05). However, the total hospitalization cost in LA group was significantly lower than that in GA group (*P *< 0.05). Further analysis of different ages in the two groups showed that there were significant differences in the VAS for back pain on the second day postoperatively and ODI at 1 week postoperatively in the middle-aged group (45 ≤ Y ≤ 59), as well as the VAS for back pain on the second day postoperatively in the senior group (Y ≥ 60) (*P *< 0.05). However, there were no significant difference among other groups (*P *> 0.05).

**Conclusion:**

Long-term outcomes were similar for both PTED under LA and GA, while GA group had better short-term outcomes, especially in middle-aged and elderly patients.

## Introduction

Lumbar disc herniation (LDH) is becoming more and more common as people change their lifestyles. When conservative treatment fails and the condition progresses, surgery may be indicated. Percutaneous transforaminal endoscopic discectomy (PTED) is a minimally invasive technique for LDH that is comparable to open surgery and microendoscopic lumbar discectomy in terms of efficacy. At the same time, it has the advantages such as a tiny incision, less bleeding, quick postoperative recovery, getting out of bed early and so on (1–3).

Most PTED are performed under local anaesthesia (LA). To avoid harm to the spinal cord and nerve roots, patients remain conscious throughout the treatment and can provide abnormal input to the operator concerning pain, numbness, and electrical sensations in the leg at any time. PTED under LA, on the other hand, is not without debate, given the increased desire for comfort and painlessness. LA is insufficient for pain relief, and some patients are unable to take it, resulting in complications during surgery and even the need to abandon the procedure (4, 5). Therefore, some researchers believe that general anesthesia (GA) is better for PTED, especially for patients who have a low pain threshold (6). Although PTED under GA can offer appropriate analgesia, due to full sensory blockade, the risk of surgery may be considerably enhanced (7). How to better manage pain during PTED has become a major clinical issue for spine surgeons.

As far as we know, few researches have examined the efficacy of PTED in LA or GA. Therefore, we conducted a retrospective case-control study to compare the clinical outcomes of PTED patients treated with LA vs. GA.

## Materials and methods

### Inclusion and exclusion criteria

The inclusion criteria were: (1) patients with single-segment LDH whose clinical symptoms and signs were consistent with the imaging findings. (2) PTED was conducted when conservative treatment failed for more than three months. (3) GA could be tolerated after assessment by an anesthesiologist. (4) the data and follow-up results were complete. The exclusion criteria were: (1) the segment with spondylolisthesis or instability required fusion surgery. (2) surgery was required for degenerative scoliosis. (3) other spinal diseases, such as ankylosing spondylitis, spinal tumors and tuberculosis and so on. (4) history of lumbar surgery.

### General information

All patients diagnosed with LDH and treated with PTED from August 2018 to August 2020 who met the inclusion and exclusion criteria were retrospectively included in this study. LA and GA were chosen according to the patient's preference. There were 72 cases in LA group and 36 cases in GA group. The study was approved by the hospital ethics committee and all patients were operated on by the same group of senior doctors. General data of the two groups were shown in Table 1. There were no significant differences in gender, age, course of disease, body mass index and surgical segment between the two groups (*P *> 0.05).

**Table 1:** General data of patients in the two groups.

Subjects	LA Group (*n *= 72)	GA Group (*n *= 36)	*P*
Male/Female	48/24	21/15	0.405
Age (years)	47.82 ± 15.55	48.78 ± 16.08	0.766
Course of disease (months)	15.53 ± 19.67	15.17 ± 18.25	0.928
Body mass index (kg/m^2^)	21.72 ± 2.34	21.70 ± 2.25	0.967
Surgical segment			0.199
L1-2	0	2	
L2-3	2	3	
L3-4	4	2	
L4-5	58	26	
L5-S1	8	3	

### Surgical procedure

To permeate the epidermis, 2–3 ml of 1% lidocaine was administered in LA group, followed by 8–10 ml layer by layer. When the superior articular process was reached, 2–3 ml was utilized to anesthetize the facet joints. If necessary, dosage could be increased appropriately. In GA group, experienced anesthesiologists performed anesthesia according to standardize intravenous compound endotracheal general anesthesia.

The patient was positioned prone on the operating table. The entrance location was around 12–14 cm distant from the midline. The needle had reached the medial and ventral surfaces of the superior articular process, according to fluoroscopy. Then a guidewire was used to replace the needle. A serial dilator was adopted and twisted to enlarge the subcutaneous tract. A protective tube was inserted into the intervertebral foramen and trephine (Spinendos, Munich, Germany) was introduced through the tube. After that, the trephine was utilized to do foraminoplasty. An endoscope (Elliquence, New York, USA) was connected. Then nerve root was revealed and herniated nucleus pulposus was excised endoscopically. The endoscope was removed and the operation ended.

### The assessment of clinical outcomes

Our study focused on factors including duration of operation, intraoperative bleeding, time of intraoperative fluoroscopy, length of hospital stay, total hospitalization cost, surgical satisfaction and complications. The length of hospital stay was from the day of admission to the day of discharge. Visual analog scale (VAS, ranging from 0 to 100, with higher scores indicating more back pain and leg pain) (8) for back pain and leg pain and Oswestry Disability Index (ODI, ranging from 0 to 100, with higher scores indicating more disability) (9) were recorded preoperatively, 1 week postoperatively and 1 year postoperatively. On the second day after surgery, the patients were asked about their satisfaction with the operation and answered “satisfactory”, “average” and “unsatisfactory”. Patients were followed up for reoperation at 1 year postoperatively, and surgical outcomes were assessed according to MacNab criteria.

### Statistical analysis

SPSS 25.0 statistical software was used for data analysis. The quantitative data were described as means ± standard deviation ($\bar{x}$_ _± s), and the qualitative data were expressed as the number of cases. Quantitative data were compared by independent sample T-test. For those failing to meet the t-test conditions, rank sum test was used. Qualitative data were compared by χ2 test. *P *< 0.05 was considered statistically significant.

## Results

All patients underwent surgery successfully. The comparison of clinical outcomes between the two groups was shown in Table 2. There were no significant differences in duration of operation, intraoperative bleeding, time of intraoperative fluoroscopy, length of hospital stay, surgical satisfaction and complications between the two groups (*P *> 0.05). However, the total hospitalization cost of LA group was significantly lower than that of GA group (*P *< 0.05). One patient in each group was reoperated for recurrence of the operated segment at one year postoperative follow-up (*P *> 0.05). Meanwhile, there was no significant difference in efficacy assessment of Macnab criteria between two groups (*P *> 0.05). Transient paresis occurred in five and three patients in the LA and GA groups, respectively.

**Table 2:** Comparison of clinical outcomes between the two groups.

Subjects	LA Group (*n* = 72)	GA Group (*n* = 36)	*P*
Duration of operation (minutes)	94.10 ± 33.21	96.94 ± 33.64	0.677
Intraoperative bleeding (ml)	9.79 ± 4.55	10.75 ± 6.02	0.358
time of intraoperative fluoroscopy (times)	25.75 ± 7.13	22.75 ± 8.05	0.063
Length of hospital stay (days)	6.13 ± 2.47	6.22 ± 2.27	0.843
Total hospitalization cost (RMB)	34,018.5 ± 7259.26	44,715.54 ± 21,656.04	<0.001
Reoperation	1 (1.39%)	1 (2.78%)	0.614
Satisfaction of surgical			0.082
Satisfactory	57	32	
Average	15	3	
Unsatisfactory	0	1	
Macnab satisfaction			0.858
Excellent	37	18	
Good	31	15	
Fair	4	3	
Poor	0	0	
Transient paresis	5	3	0.448

**Figure 1:**
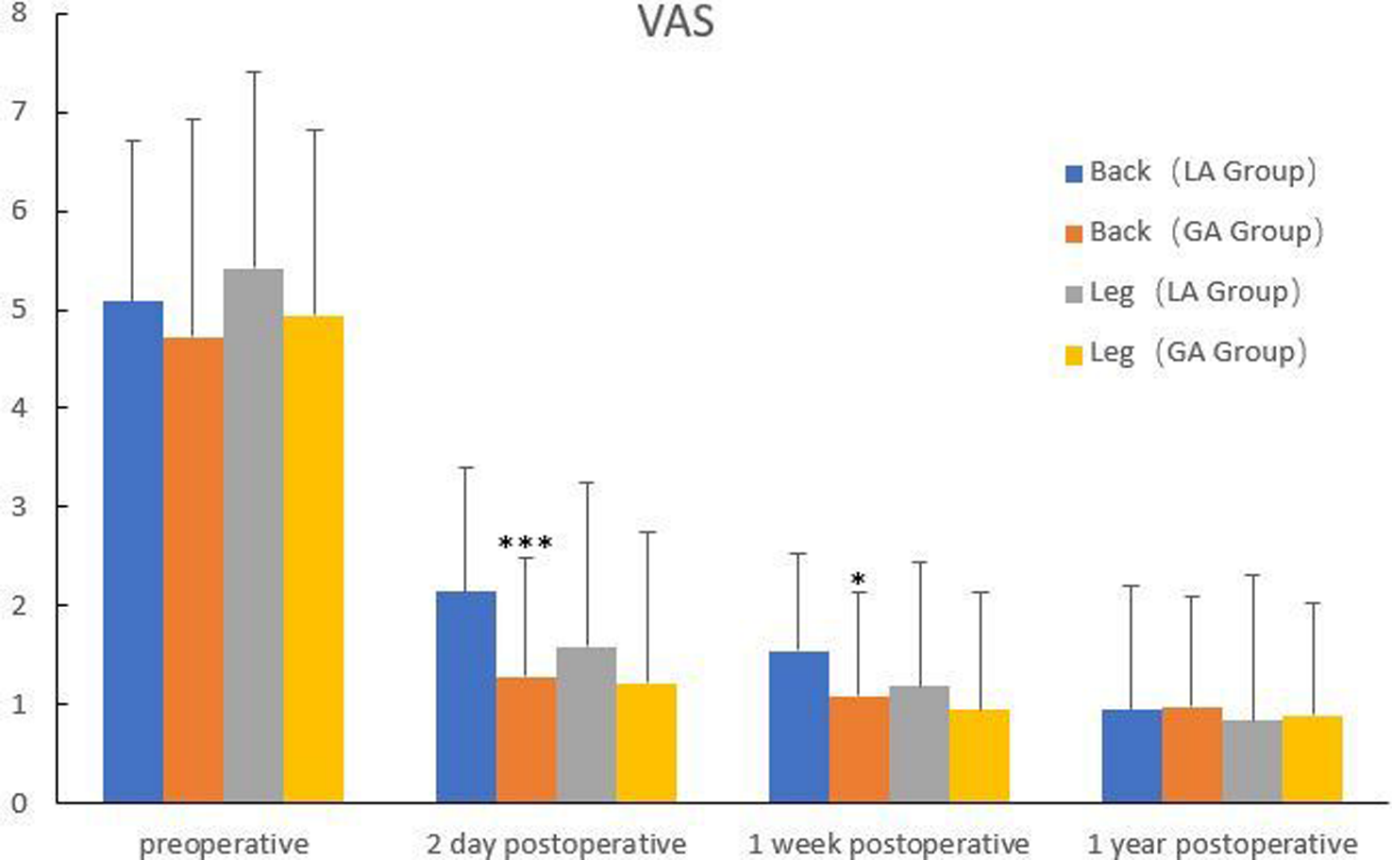
Comparison of VAS between the two groups. ***Compared with the back of LA group on the second day postoperatively, *P*<0.001. *Compared with the back of LA group at one week postoperatively, *P*<0.05.

**Figure 2:**
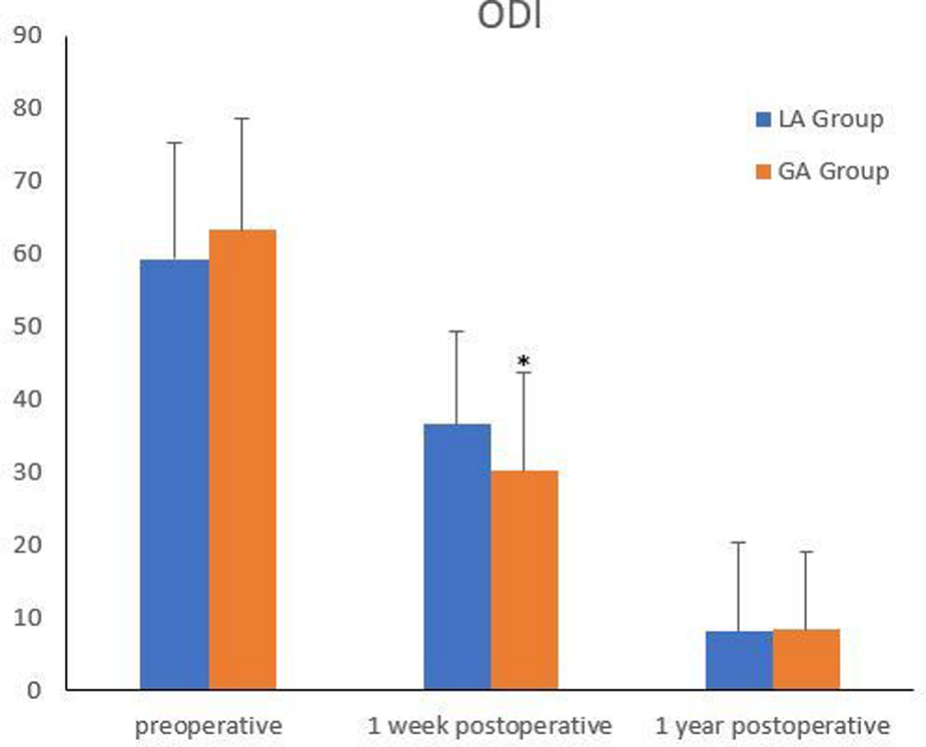
Comparison of ODI between the two groups. *Compared with the LA group at one week postoperatively, *P*<0.05.

The comparison of efficacy between the two groups was shown in Figures 1, 2. There were no significant differences in preoperative VAS for back pain and leg pain and ODI between the two groups (*P *> 0.05). Although there was no statistical difference in VAS for leg pain between the two groups on the second day after surgery (*P *> 0.05), VAS for back pain of GA group was markedly better than that of LA group (*P *< 0.05). One week after surgery, VAS for back pain and ODI in GA group were better than those in LA group (*P *< 0.05), but there was no significant difference in VAS for leg pain between the two groups (*P *> 0.05). There were no significant differences in VAS for back pain and leg pain and ODI between the two groups at 1 year follow-up (*P *> 0.05).

**Figure 3:**
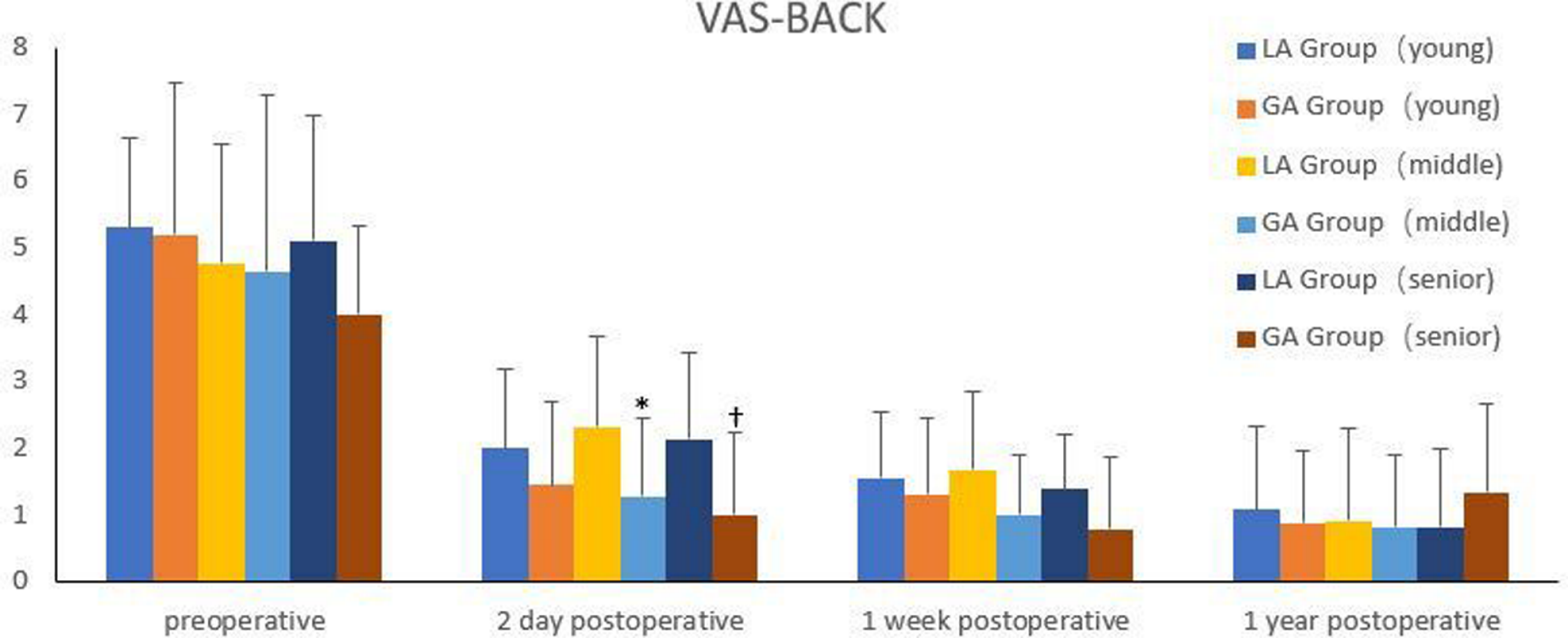
Comparison of VAS for back pain between the two groups in different age groups. *Compared with the LA group on the second day postoperatively in the middle-aged group, *P*<0.05. ^†^Compared with the LA group on the second day postoperatively in the senior group, *P*<0.05.

**Figure 4:**
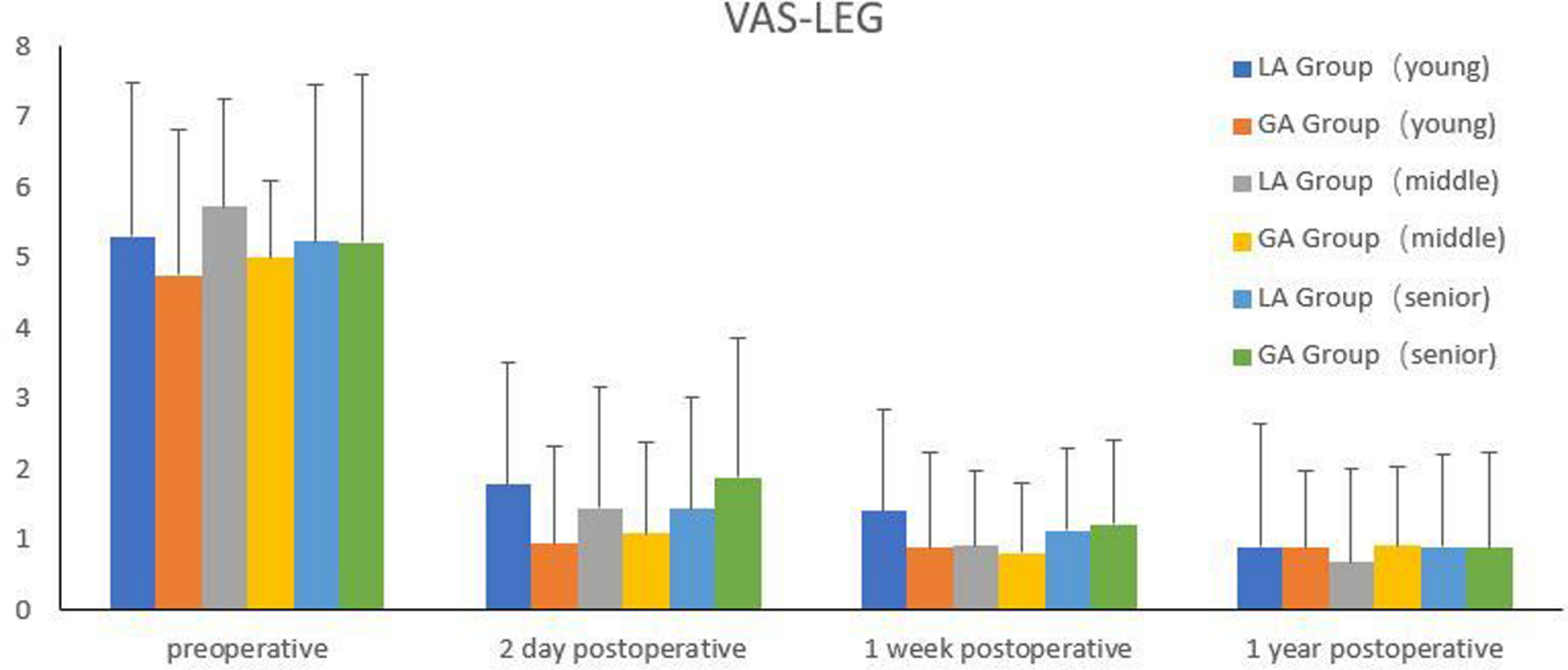
Comparison of VAS for leg pain between the two groups in different age groups.

**Figure 5:**
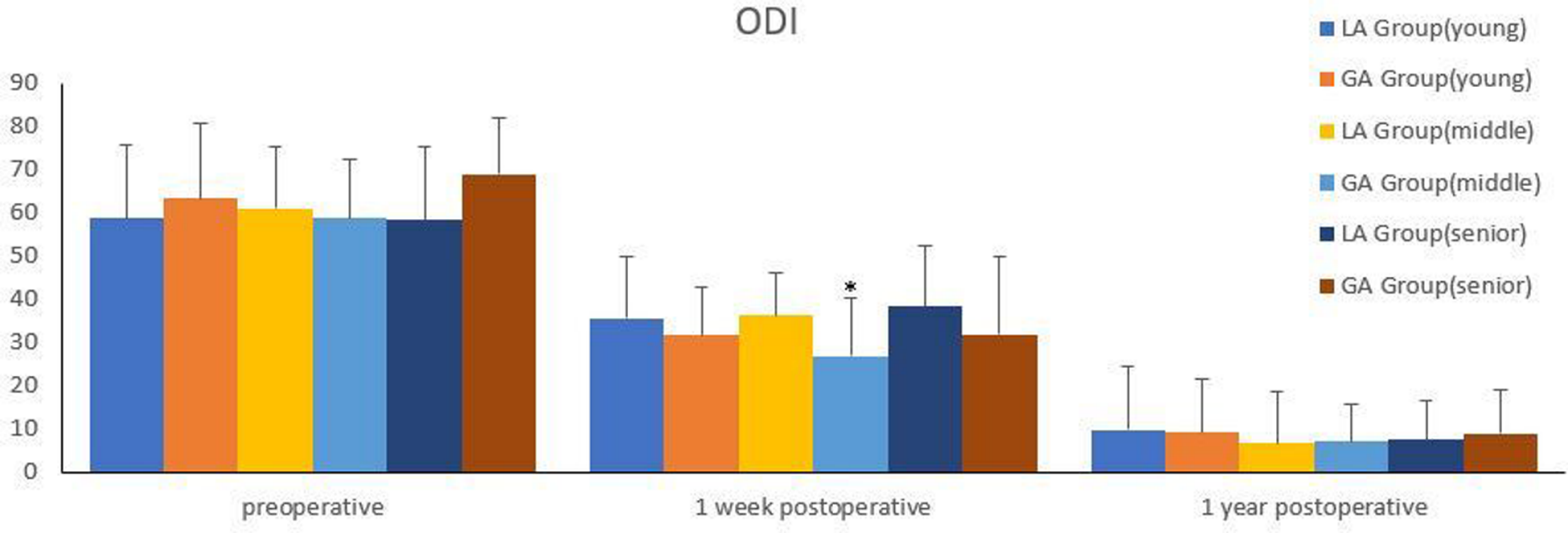
Comparison of ODI between the two groups in different age groups. *Compared with the LA group at one week postoperatively in the middle-aged group, *P*<0.05.

All the patients included were separated into three groups, according to WHO age classification criteria (10). The young group was under 45 years old; the middle-aged group was 45–59 years old; and the senior group was over 59 years old. Comparison of efficacy between GA group and LA in different age groups was shown in Figures 3–5. There were significant differences in the VAS for back pain on the second day postoperatively, ODI at one week postoperatively in the middle-aged group, as well as the VAS for back pain on the second day postoperatively in the senior group (*P *< 0.05). However, there were no significant difference among other groups (*P *> 0.05).

## Discussion

It is difficult to puncture and implant the working channel under direct vision in PTED, and there is a risk of nerve irritation during the operation. As a result, it's critical to keep the patient conscious during the procedure. Currently, most surgeons utilize LA for PTED, but some employ epidural anesthesia, lumbar anesthesia or GA. Yu et al. (11) discovered that PTED performed under local anesthesia with 0.5% lidocaine was lesser invasion, shorter hospital stays, quicker pain relief, and functional recovery compared to microendoscopic discectomy under general anesthesia. Zhang et al. (12) concluded that epidural anesthesia with low-concentration ropivacaine and sufentanil is safe and effective for PTED. Wang et al. (13) found that PTED and percutaneous endoscopic interlaminar discectomy under general anesthesia were equally cost-effective and valuable interventions for L5-S1 lumbar disc herniation. Our study showed that duration of operation, intraoperative bleeding, time of intraoperative fluoroscopies, length of hospital stay, reoperation, surgical satisfaction, Macnab satisfaction, complications and long-term outcomes were similar between LA and GA groups. Although the efficacy of GA group was better than that of LA group on the second day and 1 week postoperatively, the total hospitalization cost was higher than that of LA group.

PTED can be performed under LA because it exerts little damage to tissue. The surgeon can completely communicate with the patient during the procedure, reducing the danger of nerve injury. Meanwhile, LA offers minimal risk and low cost. As a result, LA is frequently suggested in clinical settings. However, we discovered that pain management under LA was ineffective. This could be related to the large amount of nerve fibers in the tissues surrounding the lumbar joints, which are difficult to totally block. Especially in the process of establishing working channels, foraminoplasty and releasing adherent nerve roots, severe pain is often produced, which is consistent with the study of Zhu (14). Due to the painful operation under LA, patients may have anxiety and fear about it, which may reduce the satisfaction of the surgery, so that patients may refuse to accept it again. This could have a negative impact on the promotion of PTED. In addition, this study also found that intraoperative muscle tension would limit the operation of endoscopic instruments, prolong the duration of operation, and increase the dose of radiation and surgical difficulty. Pain management that is effective can increase clinical outcomes and patient satisfaction (15).

Although there was no significant difference in the number of fluoroscopies between the two groups in this study, we did find that GA group had a slightly lower frequency than LA group, which could be due to the intraoperative analgesic effect (16). In LA group, muscle tension and postural changes might occur because of pain, which might affect the fluoroscopic effect. The frequency of fluoroscopy is closely related to the patient's coordination. In LA group, patients might ask the surgeon to stop the puncture and insertion of the working channel due to unbearable pain. Besides, the patient could move autonomously during the operation, and muscle tension due to fear might increase the frequency of fluoroscopy. The International Commission on Radiological Protection (ICRP) also recommends annual radiation limits, and the repeated fluoroscopy during surgery is too damageable to ignore (17). Our study showed that although the long-term outcomes of the two groups was consistent, the VAS for back pain on the second day postoperatively, the VAS for back pain and ODI at one week postoperatively in GA group were better than those in LA group. We may consider the following two reasons: On the one hand, patients in the GA group cooperated better during the procedure. As a result, the surgeon would be able to perform better, removing more nucleus pulposus and better releasing the nerve root. After surgery, the GA group, on the other hand, had no bad memories and felt better about themselves. Therefore, it is an option to operate PTED under GA. To reduce nerve injury, the assistant can be asked to touch the ipsilateral leg during the operation. The operation was suspended, and the position was adjusted in time when the leg beating appeared. However, owing to the steeper learning curve of PTED, it is still recommended to perform it under LA in the early stage (18). If the situation allows, it can be done under the supervision of PTED-trained surgeons to assure the surgery's safety. As experience we gain, we can transition to GA. It is good for postoperative recovery when the patient is undergoing painless surgery.

Further examination of the two age groups revealed that there was no significant difference between the young and the older groups. However, in middle-aged and older people, short-term outcomes in the GA group were better than those of the LA group. It could be linked to the degree of degeneration in middle-aged and elderly people. As they have a lower pain tolerance than young people, their postoperative back discomfort is more noticeable (14). Therefore, young people can have a variety of anesthesia options, more inclined to LA. GA is more suitable to the elderly. In addition, as our findings revealed, the total hospitalization cost in the GA group was significantly higher than in the LA group, amounting to approximately 10,697 RMB, due to the need for full participation of anesthesiologists. In terms of complications, both groups of patients suffered transitory paresis, which was assumed to be related to mechanical nerve root extraction. To avoid injury the nerve, we should carefully examine the radiography before surgery, measure the size of the foramen, and then determine the puncture direction and angle. Hussain (19) reported that PTED under GA, supplemented by neuro electrophysiological monitoring, could preferably ensure the safety of spinal cord and nerve root.

Although the aforementioned findings are clinically significant, there still exist flaws. To begin with, this was a retrospective study with a selective bias in data gathering. Second, because all of the cases came from a single center, the total number of cases was insufficient. Finally, there was no follow-up on mid-term results after surgery in the study. As a result, future research should include a comparison of the impacts at multiple time periods, as well as a prospective, large-sample multicenter cohort study to confirm our findings.

## Conclusion

Both PTED under LA and GA are safe and effective for treating patients with LDH in Long-term outcomes,while GA group had better short-term outcomes, especially in middle-aged and elderly patients. Therefore, GA can be considered a feasible alternative to LA for PTED.

## Data Availability

The original contributions presented in the study are included in the article/Supplementary Material, further inquiries can be directed to the corresponding author/s.
